# Purification of Parietal and Chief Cells from the Gastric Mucosa

**DOI:** 10.1100/tsw.2002.853

**Published:** 2002-06-15

**Authors:** John Graham

**Affiliations:** School of Biomolecular Sciences, Liverpool John Moores University, UK

**Keywords:** gastric mucosa, parietal cells, chief cells, ion transport, OptiPrep^TM^, iodixanol, discontinuous gradient

## Abstract

Acid-secreting parietal cells from the gastric mucosa are widely studied as a model in studies on ion transport. A discontinuous gradient of iodixanol has been found to be superior to earlier protocols using Nycodenz® and this method, which removes a significant amount of contaminating cells and mucus is a very useful prelude to further purification by elutriation.

**METHOD TYPE:** extraction, isolation, purification and separation

**SUB METHOD TYPE:** centrifugation

## INTRODUCTION

Investigations into the functioning and control of cells of the gastric mucosa are important in understanding the physiological activity of this tissue in both normal and clinical conditions. They have a broader significance, however, since the gastric mucosa is widely recognized as an important model system for studying the functioning and control of any secretory epithelium.

Probably the most intensively studied cell is the acid-secreting parietal cell as this provides a very useful model for studying both the regulation of ion-transport and intracellular signaling pathways[1]. ECL cells are the main endocrine/paracrine cell type and they play an important role in controlling acid secretion[2]. The other major cell type is the chief cell that secretes pepsin.

Since the late 1980s the most widely used strategy is to purify parietal cells from Pronase/ collagenase-disaggregated gastric mucosa (from both rats and rabbits) on either continuous[3,4,5] or discontinuous[5] Nycodenz® gradients, followed by centrifugal elutriation.

More recently Nycodenz® has been replaced by OptiPrep™[1] as the latter gave improved purities (80–90%) and for some studies this may be a sufficient degree of purification without the need to carry out a subsequent elutriation step (see Note 1).

The Protocol Article (adapted from Refs. [1] and [5]) addresses only the density gradient step since methods for enzymic disaggregation of the mucosa will already be established in the laboratory or can be found in Refs. [1], [3], [4], and [5].

A companion Protocol Article addresses the purification of ECL cells[6].

## MATERIALS AND EQUIPMENT

OptiPrep™ (60% w/v, iodixanol)

Additive Solution: 40 mg/ml bovine serum albumin (BSA), 2.0 mM DTT, 9.6 mM KCl, 4.8 mM $MgSO_4$, 60 mM Hepes

OptiPrep™ Diluent: mix 10 ml of 10x Hank's Buffered Salt Solution (containing Ca and Mg) with 50 ml of Additive Solution; adjust to pH 7.4 with 1.0 M Tris and make up to 100 ml with water

OptiPrep™ Working Solution (WS) of 24.4% (w/v) iodixanol ($\rho$ = 1.134 g/ml): mix 9 vol. of OptiPrep™ with 11 vol. of OptiPrep™ diluent and 2 vol. of water (see Note 2).

WS Diluent: 10 mg/ml BSA, 132 mM NaCl, 5.4 mM KCl, 1.2 mM $MgSO_4$, 0.5 mM DTT, 15 mM Hepes, adjusted to pH 7.4 with 1.0 M Tris

Cell Suspension Medium (CSM): 114.4 mM NaCl, 5.4 mM KCl, 5 mM $Na_2HPO_4$, 1 mM $NaH_2PO_4$, 1.2 mM $MgSO_4$, 1 mM $CaCl_2$, 0.01 mg/ml phenol red, *10 mM glucose, 1 mM pyruvate, 2 mg/ml BSA, 0.5 mM DTT, 0.1 mM Hepes* (add the italicized reagents immediately before use) and buffer with NaOH to pH 7.4

Plastic conical centrifuge tubes (12–15 ml)

Syringe with metal cannula (for underlayering) and/or plastic Pasteur pipette (for overlayering)

Low-speed (temperature-controlled) centrifuge with swinging-bucket rotor

## METHOD

1. Prepare gradient solutions of $\rho$ = 1.134, 1.091, 1.070, and 1.048 g/ml by diluting WS with WS Diluent; these densities are equivalent to iodixanol concentrations of 24.4, 16.2, 12.2, and 8.1%, respectively (see Note 3).
2. In a centrifuge tube, layer 2 ml each of the four gradient solutions and allow to form a continuous gradient by diffusion (see Notes 4 and 5).
3. After the mucosa has been digested enzymically and filtered, the single cell suspension is washed twice and resuspended in CSM (0.1–0.15 ml of packed cells per ml) using low speed centrifugation.
4. Layer 2 ml of the cell suspension over the linear iodixanol gradient and centrifuge at 1,000*g* for 8 min at 20–22°C (see Notes 1).
5. The banding of the cells is shown in Fig. 1.
6. Harvest band A; dilute with 2 vol of WS Diluent; harvest by centrifugation at 1,000–2,000*g* for 1 min and resuspend as required.

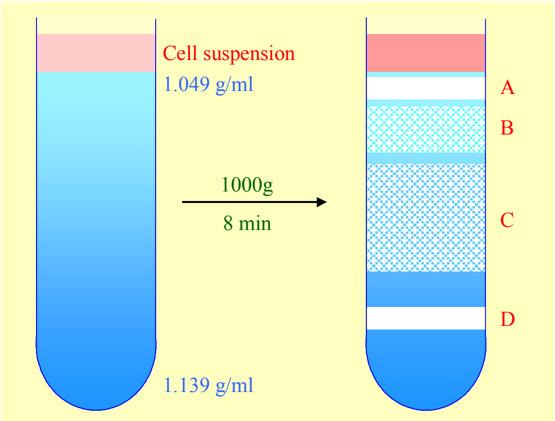

**FIGURE 1**. Fractionation of gastric mucosal cells in a continuous iodixanol gradient[1]. Band A contains 80–90% parietal cells; band B is a diffuse band of less pure parietal cells; band C is a mixed cell/mucus band, and band D contains chief cells

## NOTES

1. Parietal cells are clearly among the least dense of a mixed population of predominantly denser cells. In this respect they are not unlike peripheral blood monocytes, pancreatic Islets of Langerhans, dendritic cells from spleen, etc. Flotation methods for isolating the least dense cell type have been notably more successful in purifying these cells than sedimentation methods (e.g., [7]). It is possible that flotation of parietal cells may provide a purer isolate than sedimentation and avoid subsequent elutriation.
2. Check the pH is 7.4 before adding the water.
3. If, for operational reasons, the composition of the Additive Solution and/or Optiprep™ and WS Diluents need to be different from those given, it may be necessary to adjust the concentration of iodixanol in the gradient solutions to provide the correct density. However, as long as these solutions are based on some sort of isotonic balanced salt solution (density approx 1.006 g/ml) the iodixanol concentrations should be valid.
4. Methods for describing the formation of continuous gradients from discontinuous gradients by diffusion or by using a two-chamber gradient mixer or Gradient Master® are described Ref. [8].
5. Chew[5] reported that a discontinuous gradient of Nycodenz® can also be effective in purifying parietal cells, by implication this is probably also true of iodixanol gradients.

## ACKNOWLEDGEMENTS

The author and TheScientificWorld wish to thank Axis-Shield PoC, AS, Oslo, Norway for their kind permission to adapt OptiPrep™ Application Sheet C28 in the preparation of this Protocol Article and Professor Catherine Chew, Professor of Medicine, Cellular Biology and Anatomy, Medical College of Georgia, Augusta, GA 30912 for her assistance in the preparation of the protocol.

## References

1. Chew, C.S., Parente, J.A., Chen, X., Chaponnier, C., and Cameron R.S. (2000) *J. Cell Sci.* **113,** 2035–2045.
2. Lindström, E., Lerner, U.H., and Håkanson, R. (2001) *Eur. J. Pharmacol.* **416**, 255–263.
3. Chew, C.S. and Brown, M.R. (1986) *Biochim. Biophys. Acta* **888,** 116–125.
4. Le Goascogne, C., Sananes, N., Eychenne, B., Gouezou, M., Baulieu. E.-M., and Robel, P. (1995) *Endocrinology* **136**, 1744–1752.
5. Chew, C.S. (1990) *Meth. Enzymol.* **191**, 640–661.
6. Graham, J.M. (2002) Purification of ECL cells from gastric mucosa. *TheScientificWorldJOURNAL* **2**, in press.
7. Graham, J.M. (2002) Fractionation of hepatic nonparenchymal cells. *TheScientificWorldJOURNAL* **2**, 1347–1350.
8. Graham, J.M. (2002) Preparation of pre-formed iodixanol gradients. *TheScientificWorldJOURNAL* **2**, 1351–1355.
